# Genetic admixture and heterosis may enhance the invasiveness of common ragweed

**DOI:** 10.1111/eva.12445

**Published:** 2016-12-20

**Authors:** Min A. Hahn, Loren H. Rieseberg

**Affiliations:** ^1^ Department of Botany and Biodiversity Research CentreUniversity of British ColumbiaVancouverBCCanada; ^2^ Department of BiologyIndiana UniversityBloomingtonINUSA

**Keywords:** *Ambrosia artemisiifolia*, biological invasion, common ragweed, genetic admixture, heterosis, intraspecific hybridization, outbreeding depression

## Abstract

Biological invasions are often associated with multiple introductions and genetic admixture of previously isolated populations. In addition to enhanced evolutionary potential through increased genetic variation, admixed genotypes may benefit from heterosis, which could contribute to their increased performance and invasiveness. To deepen our understanding of the mechanisms and management strategies for biological invasions, we experimentally studied whether intraspecific admixture causes heterosis in common ragweed (*Ambrosia artemisiifolia*) by comparing the performance of crosses (F1) between populations relative to crosses within these populations for each range (native, introduced) under different ecologically relevant conditions (control, drought, competition, simulated herbivory). Performance of admixed genotypes was highly variable, ranging from strong heterotic effects to weak outbreeding depression. Moreover, heterosis was not uniformly observed among between‐population crosses, but certain native population crosses showed considerable heterosis, especially under simulated herbivory. In contrast, heterosis was largely absent in crosses from the introduced range, possibly implying that these populations were already admixed and benefit little from further mixing. In conclusion, these results support the hypothesis that heterosis may contribute to biological invasions, and indicate the need to minimize new introductions of exotic species, even if they are already present in the introduced range.

## 1 Introduction

With enhanced global trade and transport over the past several centuries, the number of species that have either intentionally or accidentally become introduced into new regions has dramatically increased. Some of these exotics thrive in their new ranges and can cause serious problems for the environment, agriculture or human health (Pimentel, Lach, Zuniga, & Morrison, 2000; Sakai et al., 2001; Vitousek, DAntonio, Loope, Rejmanek, & Westbrooks, 1997). Strategies for the management and control of current invasions, as well as the prevention of future biological invasions, may be aided by a deeper understanding of the processes and mechanisms that underlie invasion success. While early research on this question focused mainly on ecological aspects of invasions (Keane & Crawley, 2002; Levine, Adler, & Yelenik, 2004), the role of evolutionary changes in invasions has increasingly gained attention (Blossey & Nötzold, 1995; Lee, 2002; Müller‐Schärer, Schaffner, & Steinger, 2004; Prentis, Wilson, Dormontt, Richardson, & Lowe, 2008). But, despite significant advances in this field in recent years, a number of unresolved questions concerning the genetic processes associated with invasions remain (Bock et al., 2015).

Due to the strong connection between species introductions and global trade and transport, it is not surprising that many invaders have become introduced to new regions multiple times (Bossdorf et al., 2005; Dlugosch & Parker, 2008). In addition to enhanced propagule pressure, which increases the likelihood that an introduced species will persist (Simberloff, 2009), the introduction of individuals from genetically differentiated source populations and subsequent genetic admixture may have important consequences for invasion success (Rius & Darling, 2014; Verhoeven, Macel, Wolfe, & Biere, 2011). In the long term, invaders may benefit from increased genetic variation, which can reduce negative effects of genetic bottlenecks and drift, and facilitate rapid adaptation of introduced populations to novel conditions (Lavergne & Molofsky, 2007). On a shorter timescale, genetically admixed individuals may benefit from heterosis (hybrid vigor), that is, the phenotypic superiority of hybrid genotypes compared to their parents (Lippman & Zamir, 2007), which may contribute to the often observed increased performance of introduced genotypes relative to genotypes from the native range when compared in common gardens (Blossey & Nötzold, 1995; Bossdorf et al., 2005).

Three main genetic models have been proposed to explain the increased performance of newly formed hybrids: 1) the dominance hypothesis, which attributes heterosis to the masking (complementation) of undesirable recessive alleles from one parent by desirable dominant alleles from the other parent, 2) overdominance, which refers to the enhanced performance of heterozygous genotypes compared to homozygotes at a given locus, and 3) epistasis, which ascribes heterosis to complex interactions between genes (Hochholdinger & Hoecker, 2007; Lippman & Zamir, 2007). According to these models, heterosis is expected to be maximal in crosses between strongly differentiated and presumably inbred populations. However, analogous mechanisms could also reduce the fitness of hybrids, leading to outbreeding depression. This may be the case, for example, if genetic admixture results in genetic incompatibilities, underdominance or the loss of local adaptation through the introduction of maladapted alleles, or the breakup of adapted gene complexes (Lynch, 1991).

There is increasing empirical evidence that genetic admixture and heterosis may play important roles during biological invasions (Rius & Darling, 2014), with important implications for management and control strategies. Several population genetic studies have revealed mixed ancestries of invasive populations reflecting genetic admixture of multiple divergent native source populations (e.g., Kolbe et al., 2004; Rosenthal, Ramakrishnan, & Cruzan, 2008; Chun, Fumanal, Laitung, & Bretagnolle, 2010; Stephen R. Keller, Gilbert, Fields, & Taylor, 2012). Moreover, some studies also provide evidence for phenotypic changes and effects on fitness associated with admixture (Kolbe, Larson, & Losos, 2007; Facon, Pointier, Jarne, Sarda, & David, 2008; S. R. Keller & Taylor, 2010). However, although these observational studies suggest a strong link of genetic admixture and invasion success, it remains difficult to disentangle the direct effects of genetic admixture (e.g., heterosis) from long‐term effects of increases in genetic variation (evolutionary potential), as well as other confounding effects such as propagule pressure that may be associated with multiple introductions. So far, only a few experimental studies have addressed these questions. For example, Turgeon et al. (2011) showed that experimental crosses of the invasive harlequin ladybird *Harmonia axyridis* benefited from admixture of different source strains, which may have contributed to the invasiveness of this species. Likewise, in a recent study, Van Kleunen, Roeckle, and Stift (2015) found increased fitness in crosses between different populations of the invasive plant *Mimulus guttatus* compared to within‐population crosses, in particular in crosses between native and invasive populations. While these studies provide important mechanistic insights into the role of genetic admixture and heterosis for invasions, more experimental work is needed to assess the generality of these findings. Theoretical questions of particular interest include the relative importance of positive (i.e., heterosis) vs. negative (i.e., outbreeding depression) outcomes of admixture in invasions, the expression of heterosis under different environmental conditions, and the contributions of selection toward preserving heterotic effects in subsequent generations. From a practical perspective, a better understanding of the role of heterosis in invasions is critical for guiding management efforts (i.e., prevention of new introductions and gene flow, monitoring admixture in introduced species) and to improve control options (i.e., evaluating the efficacy of control measures).

In this study, we used an experimental approach to address these questions in *Ambrosia artemisiifolia* L. (common ragweed, Asteraceae), which is one of the most problematic plant invaders in Europe and in several other regions of the world. It is difficult to control because of its large seed production, resulting in yield losses in crop fields (Cowbrough, Brown, & Tardif, 2003), as well as major human health problems because of its highly allergenic pollen (Laaidi, Laaidi, Besancenot, & Thibaudon, 2003). From its native range in North America, *A. artemisiifolia* has been inadvertently introduced to Europe as a seed contaminant. Previous population genetic studies provide clear evidence for multiple introductions and genetic admixture in European populations of *A. artemisiifolia* (Chun et al., 2010; Gaudeul, Giraud, Kiss, & Shykoff, 2011; Genton, Shykoff, & Giraud, 2005). In addition, invasive genotypes have been found to show increased performance compared to native genotypes, which suggests that evolutionary changes may underlie the invasion success of this species (Hodgins & Rieseberg, 2011). Given the high levels of genetic admixture reported in this species, as well as the large effects of heterosis commonly observed in many crop species (Schnable & Springer, 2013), we hypothesized that heterosis may have contributed to the increased performance of invasive genotypes of *A. artemisiifolia* and could play a key role for the invasion success of this species.

To deepen our understanding on the potential mechanistic role of heterosis in the invasion of *A. artemisiifolia* as well as its implications for management and control, we studied the outcomes of admixture in experimentally reconstructed admixed genotypes (between‐population crosses) from putative native source populations by estimating heterosis based on their relative performance to nonadmixed genotypes (within‐population crosses) from the same populations. In addition, we created admixed genotypes from the introduced range, which allowed us to evaluate differences in heterosis between crosses from presumably highly differentiated native populations as compared to crosses from less differentiated, admixed populations from the introduced range. Furthermore, we studied the expression of heterosis under a range of environmental conditions (control, drought, competition, simulated herbivory), which may be relevant for the invasion under current and future conditions, as well as to inform specific management and control options. Specifically, we asked (1) whether plants of *A. artemisiifolia* show evidence for heterosis or outbreeding depression, (2) whether heterosis is more pronounced in the crosses between native populations than those from the invaded range, (3) whether the level of heterosis varies among the particular population cross‐combinations, and (4) whether the expression of heterosis or outbreeding depression differs among environmental conditions.

## 2 Materials and Methods

### 2.1 Population sampling

**Figure 1:**
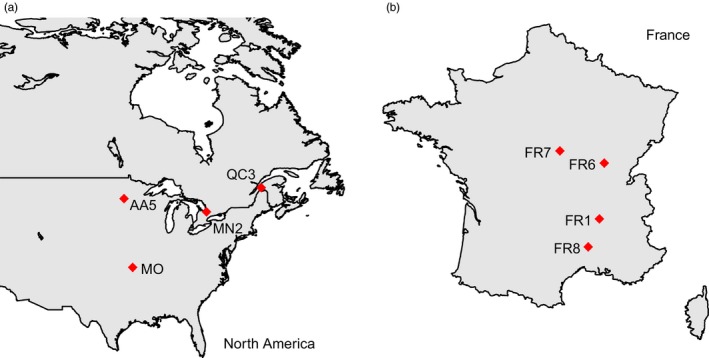
Sampling locations of the populations of *Ambrosia artemisiifolia* used in the experiment from (a) the native range (North America) and (b) the introduced range (France)

We used seed material collected from North American populations in the fall 2008 and 2013 and from European populations in the fall 2008. We selected four populations from each range (Table 1, Figure 1). The selected European populations are located across France covering a region in Europe where *A. artemisiifolia* is especially invasive, and the populations from North America are from regions that have previously been identified as putative source regions of western European populations (Gaudeul et al., 2011).

**Table 1:** Description of the populations of *Ambrosia artemisiifolia* used in the experiment (population code, location, geographical coordinates, and year of collection)

Population	Range	Country	State/Province	*N*	*W*	Year
AA5	Native	USA	MN	46.217083	−96.050194	2008
MO	Native	USA	MO	37.00644	−94.35011	2013
MN2	Native	Canada	ON	44.44716	−79.80385	2013
QC3	Native	Canada	QC	47.67876	−69.022	2013
FR7	Introduced	France	–	47.175819	3.014628	2008
FR6	Introduced	France	–	46.800028	4.972428	2008
FR1	Introduced	France	–	45.080225	4.757443	2008
FR8	Introduced	France	–	44.216656	4.264008	2008

### 2.2 Production of crosses

We produced crosses between individuals from different populations (between‐population crosses) as well as from the same populations (within‐population crosses) with four different populations or combinations of populations (“population combination” in the following) used in the within‐population and between‐population crosses, respectively. Notably, not all crosses from the possible population combinations have been produced, but only the four crosses between the geographically most distant populations in each range. From each population, seeds from three different seed families were used to obtain three independent biological replicates for each cross. The seeds were stratified for a period of 8 weeks following the procedures suggested by Willemsen (1975) starting in April 2014. In June 2014, the seeds were germinated on damp filter paper in Petri dishes with 1% plant preservative mixture in a growth chamber with a 24°C day and 18°C night and a 14:10‐hr light/dark cycle. After 2 weeks, on June 23, the emerged seedlings were transplanted into seedling trays (2.5 × 2.5 cm compartments) with a 1:1 mixture of potting soil and sand under ambient conditions in the horticultural greenhouse at the University of British Columbia. On July 15, pairs of plants of similar size for each planned cross were planted into single pots (15 cm diameter, 18 cm height) with the same soil–sand mixture. On August 5, the plant pairs were covered with pollen‐proof pollination bags (PBS International, UK) to prevent cross‐contamination of pollen from nontarget individuals. The bags were shaken every few days to assure cross‐pollination of the plants within the bags. As previous studies reported strong self‐incompatibility mechanisms in *A. artemisiifolia* (Friedman & Barrett, 2008), selfing was expected to be negligible. The mature seeds were collected between December 1, 2014 and March 27, 2015. After excluding crosses that did not set seed because of nonoverlapping flowering times, we ended up with a total of 68 crosses (including 28 reciprocal crosses to account for maternal effects), with roughly equal numbers of crosses for the different cross types (15 native within‐population crosses, 16 native between‐population crosses, 20 invasive within‐population crosses, 17 invasive between‐population crosses).

### 2.3 F1 experiment

To study heterosis under different ecologically relevant conditions, we grew plants from the F1 generation in a common garden greenhouse experiment under four different treatments (control, drought, competition, and simulated herbivory). Four individuals from each cross were used, each of which was subjected to a different experimental treatment, for a total of 272 plants. The seeds were stratified and germinated as described above. Germination was initiated on June 2, 2015, and the emerged seedlings were transplanted into seedling trays in the greenhouse on June 11, 2015. On June 22, 2015, the plants were transplanted into bigger pots (10 cm diameter) with a 2:1:1 mixture of potting soil : coarse forestry sand : fine industrial sand. After 3 days of acclimatization and adequate watering, the experimental treatments were initiated. Plants in the control treatment were automatically watered by flooding the greenhouse bench every other day for the first 4 weeks and daily afterward. Plants in the drought treatment were placed into trays to prevent automatic watering and received equal amounts of water when the first plants (typically the majority of plants) started wilting (~every 3 days on average). In the competition treatment, plants were under light and nutrient limitation due to the addition of the grass *Poa pratensis*, but received similar watering as the controls. To ensure a reasonably dense and high grass cover in the pots, we twice sowed 0.3 g of *Poa pratensis* seeds into the pots prior to the start of the experiment (on May 13 and June 12, 2015). For the simulated herbivory treatment, 50% of the total leaf area of all newly emerged leaves longer than 2 cm was cut every week. Moreover, after removal from the bench, the plants were sprayed with 5 mM methyl jasmonate until all leaves were soaked and left to dry before they were moved back. Methyl jasmonate is commonly used to elicit defense responses against herbivores in many plant species (McConn, Creelman, Bell, Mullet, & Browse, 1997). The watering regime was similar to the control plants. Three times during the experiment, plants of all treatments were fertilized with a minimal amount of 0.2 ml all‐purpose fertilizer (20‐20‐20N‐P‐K; Plant‐Prod Ultimate; Premier Tech Home & Garden Inc.). All plants were grown in a completely randomized design 25 cm apart, and the positions were rerandomized weekly throughout the experiment.

### 2.4 Measurements

Throughout the experiment, we measured several traits on the experimental plants related to growth and reproduction in regular intervals (weekly for most traits). But, as preliminary time series analyses (results not shown) did not reveal any significant interactions of time with cross types or treatments, we present only the results based on the final measurements (or single measurements for traits that were measured only once). The traits included the final plant height, stem diameter at the base of the stem, the number of leaves of 3‐week‐old plants, the number of branches, and the number of flower heads at the time of flowering onset. At the time of flowering onset for each plant (first opening of male flowers and release of pollen, between July 21 and October 16, 2015), the plants were harvested and the aboveground biomass was determined after drying to a constant weight at 65°C for 10 days.

### 2.5 Statistical analyses

We explicitly estimated the level of heterosis (and outbreeding depression) for each individual between‐population cross based on its performance relative to the mean of the two corresponding within‐population crosses (midparent heterosis). Heterosis was calculated as ((F1 − MP)/MP) × 100, in which F1 is the trait value in a given between‐population cross and MP is the mean of the trait values of the two corresponding within‐population crosses. The trait values of the within‐population crosses were based on averages of all crosses derived from the same parental lineage as the between‐population crosses to obtain most accurate estimations accounting for different genetic backgrounds. Differences in heterosis for each trait among crosses from different ranges (native, introduced) and treatments were analyzed using linear mixed‐effects models using the “lme” function from the “nlme” package in R (Pinheiro, Bates, DebRoy, & Sarkar, 2015) with range, treatment, and their interaction as fixed factors and population combination as a random factor. Differences in heterosis among crosses from specific population combinations for each range were analyzed using linear models with population combination, treatment, and their interaction as explanatory variables. Significance of model terms was evaluated by stepwise removal of the least significant term (interactions first) using likelihood ratio tests for mixed‐effects models and *F*‐tests for linear models. Differences among experimental groups were tested using least square means with the R package “lsmeans” (Lenth, 2015), and *p*‐values of pairwise Tukey contrasts were adjusted using Bonferroni corrections for multiple comparisons. All analyses were conducted using the statistical software R version 3.2.2 (R Core Team 2015).

## 3 Results

### 3.1 Heterosis in crosses from different ranges and treatments

**Figure 2:**
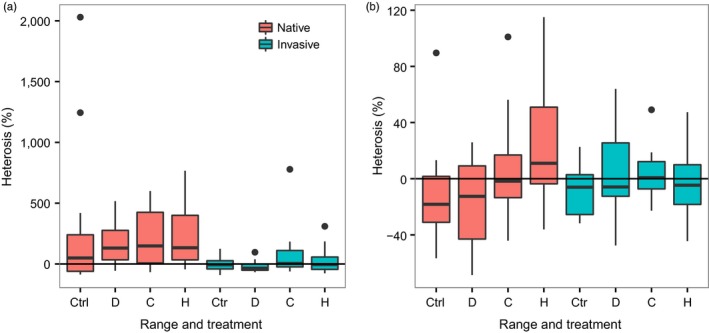
Boxplots of heterosis [%] in (a) the number of flower heads and (b) final plant height for each individual between‐population cross of *Ambrosia artemisiifolia* from the native and introduced range (native and invasive crosses, respectively) in the different experimental treatments (Ctrl: control, D: drought, C: competition, H: simulated herbivory). Heterosis estimates are based on the performance of between‐population crosses relative to the average performance of the corresponding within‐population crosses derived from the same parental lineages

The outcomes of admixture (heterosis and outbreeding depression) varied significantly among the experimental crosses from the different ranges and treatments for plant height, biomass, and the number of flower heads (Table 2). Significant heterosis (estimate bigger than zero) was only observed for crosses between native populations (Table 3, Figure 2a), which showed an average increase of 217% in the production of flower heads compared to the crosses within the corresponding native populations (Table 3). However, there was also considerable variation in levels of heterosis and outbreeding depression among individual crosses, ranging from strong heterotic effects to weak outbreeding depression (Table 3, Figure 2a). In contrast to the crosses from the native range, no significant heterosis was found for crosses between populations from the introduced range (Table 3).

**Table 2:** Effects of range (native, introduced), treatments (control, drought, competition, simulated herbivory), and their interactions on the level of heterosis in different traits of between‐population crosses of *Ambrosia artemisiifolia*. Table shows results of likelihood ratio (LR) tests of models with and without a given term, following stepwise removal of nonsignificant terms starting with interactions. Significant *p*‐Values are shown in bold

Trait	Range			Treatment			Range × Treatment		
	*df*	LR	*p*‐Value	*df*	LR	*p*‐Value	*df*	LR	*p*‐Value
Plant height	1	0.02	.8952	3	12.27	**.0065**	3	15.10	**.0017**
Stem diameter	1	0.51	.4747	3	7.47	.0585	3	5.33	.1493
Leaves	1	1.28	.2572	3	0.52	.9138	3	3.00	.3923
Branches	1	2.10	.1474	3	3.27	.3515	3	2.66	.4476
Biomass	1	0.00	.9874	3	10.20	**.0170**	3	8.50	**.0367**
Flower heads	1	18.47	**<.0001**	3	1.90	.5941	3	0.77	.8570

**Table 3:** Estimated means and standard errors of heterosis estimates (%) for different traits of between‐population crosses of *Ambrosia artemisiifolia* from different ranges (native, introduced) and treatments (control, drought, competition, simulated herbivory) from minimal adequate models. Estimates that significantly differ from zero after Bonferroni correction for multiple comparisons are highlighted in bold

Trait	Range	Treatment									
		Control		Drought		Herbivory		Competition		Overall	
		Mean	*SE*	Mean	*SE*	Mean	*SE*	Mean	*SE*	Mean	*SE*
Plant height	Native	−13.00	11.54	−17.85	11.54	25.28	11.65	5.26	11.54	–	–
	Introduced	−7.28	11.41	0.80	11.41	−3.90	11.41	2.87	11.41	–	–
Biomass	Native	−16.75	19.44	−8.48	19.44	50.95	19.71	−10.86	19.44	–	–
	Introduced	−8.68	19.15	12.78	19.15	6.74	19.15	4.61	19.15	–	–
Flower heads	Native	–	–	–	–	–	–	–	–	**217.16**	**32.51**
	Introduced	–	–	–	–	–	–	–	–	19.19	30.79

We also detected significant interactions between ranges and treatments for the estimate of heterosis in plant height and biomass (Table 2). In native crosses, heterosis for plant height was significantly higher under simulated herbivory compared to plants in the control (*df* = 118, *t* = −4.19, *p *<* *0.001) and drought treatment (*df* = 118, *t* = −4.72, *p *<* *0.0001; Table 3, Figure 2b). Also for biomass, heterosis was higher in crosses from the native range under simulated herbivory compared to plants in the control (*df* = 118, *t* = −3.70, *p *<* *0.002), drought (*df* = 118, *t* = −3.25, *p *<* *0.009), and competition treatment (*df* = 118, *t* = −3.38, *p *<* *0.006; Table 3). Again, no significant differences in heterosis were found in the crosses from the introduced range even when tested under different environmental conditions.

### 3.2 Heterosis in crosses from different population combinations and treatments

**Figure 3:**
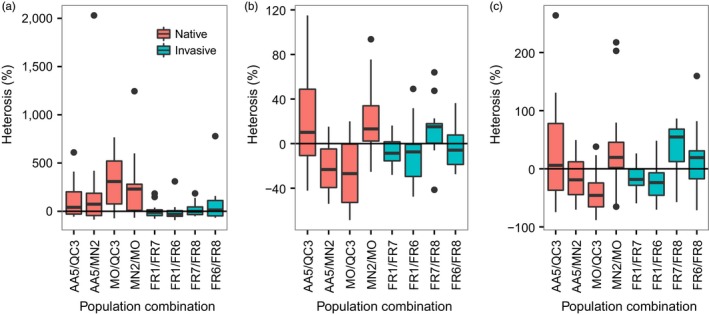
Boxplots of heterosis [%] in (a) the number of flower heads, (b) final plant height, and (c) aboveground biomass for each individual between‐population cross of *Ambrosia artemisiifolia* from different population combinations in the native and introduced range (native and invasive crosses, respectively). Heterosis estimates are based on the performance of between‐population crosses relative to the average performance of the corresponding within‐population crosses derived from the same parental lineages

The outcomes of admixture also differed significantly among population combinations and treatments (Table 4). Significant heterosis for certain population combinations was detected for most traits, but almost exclusively for crosses from the native range (Table 5). In addition, there was considerable variation in levels of heterosis and apparent outbreeding depression, which was not consistent across population combinations or traits. While crosses from all native population combinations showed heterosis for the number of flower heads (Table 5, Figure 3a), there were variable levels of heterosis and even contrasting patterns (heterosis and outbreeding depression) for plant height, stem diameter, the number of leaves, the number of branches, and biomass depending on the particular population combination (Table 5, Figure 3b,c). In contrast, the outcomes of admixture in crosses from the introduced range were less variable (Table 5, Figure 3). Moreover, the only significant case of heterosis in invasive between‐population crosses was found for biomass in the population combination FR7/FR8 (Table 5, Figure 3c), which represents the cross between the two geographically most distant populations studied from the introduced range (Table 1, Figure 1).

**Table 4:** Effects of population combinations, treatments (control, drought, competition, simulated herbivory), and their interactions on the level of heterosis in different traits of between‐population crosses of *Ambrosia artemisiifolia*. Significance of given terms was determined by *F*‐tests, following stepwise removal of nonsignificant terms starting with interactions. Separate models were fitted for crosses from the native and introduced ranges. Significant *p*‐Values are shown in bold

Range	Trait	Pop. combination			Treatment			Pop comb. × Treat.		
		*df*	*F*	*p*‐Value	*df*	*F*	*p*‐Value	*df*	*F*	*p*‐Value
Native	Plant height	3	13.42	**<.0001**	3	7.01	**<.001**	9	0.69	.7100
	Stem diameter	3	3.65	**.0175**	3	2.71	.0539	9	0.47	.8898
	Leaves	3	3.47	**.0216**	3	0.60	.6164	9	0.75	.6654
	Branches	3	6.29	**<.001**	3	0.79	.5032	9	0.53	.8484
	Biomass	3	5.50	**.0022**	3	4.41	**.0075**	9	0.79	.6266
	Flower heads	3	0.61	.6084	3	0.22	.8804	9	1.15	.3505
Introduced	Plant height	3	3.52	**.0199**	3	0.79	.5066	9	0.34	.9569
	Stem diameter	3	1.15	.3352	3	0.75	.5294	9	0.72	.6890
	Leaves	3	4.00	**.0113**	3	0.40	.7515	9	1.74	.1041
	Branches	3	0.47	.7038	3	1.48	.2283	9	1.35	.2372
	Biomass	3	7.41	**.0002**	3	0.79	.5030	9	1.47	.1825
	Flower heads	3	1.64	.1888	3	1.94	.1313	9	1.08	.3948

**Table 5:** Estimated means and standard errors of heterosis estimates (%) for different traits of between‐population crosses of *Ambrosia artemisiifolia* from different population combinations and treatments (control, drought, competition, simulated herbivory) from minimal adequate models. Estimates that significantly differ from zero after Bonferroni correction for multiple comparisons are highlighted in bold

Range	Trait	Population combination									
		AA5/QC3		AA5/MN2		MO/QC3		MN2/MO		Overall	
		Mean	*SE*	Mean	*SE*	Mean	*SE*	Mean	*SE*	Mean	*SE*
Native	Plant height	**23.04**	**8.42**	**−22.23**	**6.52**	**−24.79**	**8.42**	**23.69**	**6.70**	–	–
	Stem diameter	**24.72**	**8.10**	3.75	6.27	−13.05	8.10	3.74	6.44	–	–
	Leaves	−6.14	14.40	22.59	11.16	−24.63	14.40	26.66	11.45	–	–
	Branches	18.68	9.84	17.94	7.62	−24.28	9.84	**28.15**	**7.82**	–	–
	Biomass	31.03	17.07	−15.80	13.22	−37.87	17.07	**36.59**	**13.58**	–	–
	Flower heads	–	–	–	–	–	–	–	–	**217.16**	**44.35**

		FR1/FR7		FR1/FR6		FR7/FR8		FR6/FR8			
		Mean	*SE*	Mean	*SE*	Mean	*SE*	Mean	*SE*		
Introduced	Plant height	**−**7.72	5.30	**−**9.26	4.74	12.09	5.30	**−**2.47	5.30	–	–
	Leaves	**−18.13**	**6.12**	2.73	5.47	**−17.66**	**6.12**	2.66	6.12	–	–
	Biomass	**−**15.62	10.43	**−**20.32	9.33	**38.90**	**10.43**	13.29	10.43	–	–

## 4 Discussion

Our analyses of the effects of genetic admixture in *A. artemisiifolia* revealed large variation in the level of heterosis across different traits, crosses from the different ranges, population combinations, and environmental conditions. Interestingly, heterosis was found almost exclusively in crosses from the native range, which suggests that genetic admixture resulting from multiple introductions of differentiated native populations could have contributed to the enhanced performance of *A. artemisiifolia* genotypes from the invaded range (Hodgins & Rieseberg, 2011). The lack of heterosis in crosses from the introduced range, in contrast, may indicate that these populations are already admixed and thus no longer show significant short‐term benefits of genetic admixture.

### 4.1 Large variation in heterosis in native crosses

The immediate effects of genetic admixture between different native populations of *A. artemisiifolia* ranged from highly beneficial heterosis to weak outbreeding depression, as well as variable effects among particular population combinations, traits, and environmental conditions. Given this variation, few general patterns emerged from the data. The only consistent evidence for heterosis across all population combinations and treatments was observed in flower head production in crosses from the native range. However, certain population combinations also showed evidence of heterosis in a number of other traits, indicating that the outcomes of genetic admixture depend on context, that is, the populations, genotypes, environmental conditions, and trait of interest. This is not surprising, given the numerous possible genetic interactions during genetic admixture (Hochholdinger & Hoecker, 2007; Lippman & Zamir, 2007). While dominance, overdominance, or epistasis could result in heterosis, a similar set of mechanisms (epistasis or underdominance) can lead to outbreeding depression depending on the genotypes involved.

Studies of intraspecific crosses in other species indicate that heterosis is likely if populations have previously experienced inbreeding depression (Verhoeven et al., 2011) or are genetically differentiated (Rius & Darling, 2014). The latter prediction is in line with the large variation in performance of native populations in our study as well as previous studies showing high differentiation and significant isolation by distance among native populations of *A. artemisiifolia* (Genton et al., 2005). On the other hand, admixed genotypes can be expected to exhibit outbreeding depression due to genetic incompatibilities arising from hybridization between differentiated populations, or through the loss of local adaptation following the introduction of unfavorable alleles or the breakup of adapted gene complexes (in particular in the F2 and later generations after recombination) (Johansen‐Morris & Latta, 2006). Although in our experiment, there was some evidence for outbreeding depression, the overall pattern in our study indicates that the benefits outweighed potential negative effects of admixture in the majority of crosses.

Interestingly, the level of heterosis in the crosses from the native range in our experiment also varied among the environmental conditions with the largest effects found under presumably stressful conditions, in particular under simulated herbivory. While some effects of genetic admixture are expected to be mainly independent of the environment (e.g., reduction in inbreeding depression), others are likely to be strongly condition dependent (Rius & Darling, 2014). For example, novel gene and allele combinations resulting from hybridization between distinct parental genotypes may be better adapted to environments outside the range of conditions previously experienced by parental populations. This is seen in crop hybrids, which often show increased tolerance to stress compared to their parental lineages (Schnable & Springer, 2013). Whether these benefits eventually may contribute to the invasion success depends in part on the extent to which the environmental conditions differ between the native and introduced range (Rius & Darling, 2014). In *A. artemisiifolia*, increased growth and reproduction in introduced populations suggest that they have adapted to more competitive environments in Europe (Hodgins & Rieseberg, 2011), which is in line with the findings of our study. The high levels of heterosis observed under simulated herbivory in particular further imply that admixed genotypes may have experienced lower biotic resistance against their invasion due to increased tolerance of herbivory. This may have important implications for management strategies, as admixed genotypes may become less susceptible to control measures, such as cutting or biological control using herbivores, and therefore more difficult to control.

The large variation in outcomes of admixture observed further suggests that selection may play an important role in the subsequent evolution of admixed populations. Despite some evidence for outbreeding depression, these negative effects of intraspecific hybridization appeared to be relatively weak. This is in line with the hypothesis that the loss of local adaptation through admixture may be less crucial for introduced populations than for native populations, because they presumably lack local adaptation initially and therefore can more freely benefit from the positive effects of admixture (Verhoeven et al., 2011). Heterotic genotypes, in contrast, may be advantageous under the novel conditions and—depending on the underlying genetic mechanisms—could be fixed by selection. In the case of overdominance, heterosis results from heterozygosity, and hence, the effects are expected to be maximal in the F1 generation and will be reduced in subsequent generations due to decreasing heterozygosity. Nevertheless, even such transient effects could be beneficial for invasions, as they may assist populations in overcoming demographic challenges of small population sizes at initial stages of invasions (Drake, 2006). In contrast, if dominance or epistasis (which does not require heterozygous allele combinations) is the main mechanism of heterosis, selection may favor individuals with combinations of favorable alleles at multiple loci that increase fitness by complementing deleterious alleles (the dominance model) or through favorable epistatic interactions. Subsequently, the heterotic effects may be preserved in successive generations and potentially become fixed (Bock et al., 2015). Such heterotic gene combinations may contribute to the generation of transgressive phenotypes, which most frequently arise through the complementary action of additive loci (Rieseberg, Archer, & Wayne, 1999).

There appears to be some confusion in the invasion biology about selection on loci with dominance. While selection does not act on dominance variance, as long as a recessive allele exists at some frequency, then some of the genetic variance is additive and can be acted on by natural selection. Such additive variance can be increased in invasive populations due to founding events and population bottlenecks, potentially increasing the efficacy of selection for heterotic gene combinations (Robertson, 1952).

### 4.2 No effects of admixture in crosses from the introduced range

Strikingly, in contrast to the large variation in admixture effects in native crosses, little evidence of either heterosis or outbreeding depression was observed in crosses between populations from the introduced range. This pattern may indicate that these populations are already admixed and therefore may not obtain additional short‐term benefits from further admixture. This finding is consistent with previous studies that revealed high levels of genetic admixture and gene flow in European populations of *A. artemisiifolia*, and as a result high genetic variation within populations, but low genetic differentiation among populations (Chun et al., 2010; Genton et al., 2005).

### 4.3 Heterosis may contribute to the invasion of *Ambrosia artemisiifolia*

In summary, the findings of our study support the hypothesis that heterosis may have contributed to the invasion of *A. artemisiifolia*. Although the limited sample size in our experiment does not allow firm general conclusions, building on previous studies that reported evidence for genetic admixture (Genton et al., 2005), our work suggests that heterosis may be a frequent result of native between‐population crosses. Nevertheless, the variation we observed in admixture effects indicates that heterosis is not an invariable outcome of admixture and that selection may play an important role in maintaining the expected benefits in a population. In addition, given the few studied populations, which may not be representative for all populations and possible cross‐combinations, as well as the partial nonindependence of the cross‐combinations within each range due to our crossing design, it is likely that we have underestimated the range of outcomes as well as the potential benefits from heterosis (Kolbe et al., 2007). Moreover, admixture between populations from the native and invasive ranges, which may occur following recurrent introductions, might result in additional heterosis (Van Kleunen et al., 2015).

### 4.4 Implications for biological invasions

Our findings add to a growing body of literature suggesting that heterosis may play an important role in the success of invasions through the resulting increases in performance of admixed genotypes. Heterosis may not only be relevant in the short term, but it may also have longer‐lasting effects if heterotic gene combinations (favorable alleles at multiple loci) are fixed by selection, although evidence for the latter claim is admittedly weak. An important future goal, and beyond the scope of the present study, is to identify such heterotic genotypes and assess whether they contribute importantly to the often observed increased performance of invasive genotypes (Bossdorf et al., 2005).

In addition to these theoretical implications, our results have important practical implications, as knowledge of the potential consequences of genetic admixture is important for the prediction and prevention of biological invasions, as well as for the development of management and control options (Hulme, 2009). Most importantly, our results indicate the need to concentrate efforts on minimizing new introductions of exotic species, even if they already are present in the introduced range. In contrast, as heterosis was less apparent in crosses from the introduced range, the prevention of gene flow and admixture among populations in the introduced range may be less critical than the prevention of introductions of new genotypes from the native range. Furthermore, the elevated levels of heterosis under simulated herbivory and other stress conditions in *A. artemisiifolia* in our experiment suggest that heterosis may contribute to an increased tolerance to these conditions. Hence, admixed genotypes may be less susceptible to management and control efforts, which in turn need to be optimized. Our results further imply that also admixture among populations in a species' native range could be problematic, especially given rapidly changing environmental conditions, potentially giving rise to problematic native species (Chunco, 2014). And finally, because not all native species can be practically monitored, it probably makes the most sense to focus attention on native species such as common ragweed that are problematic and have become invasive elsewhere.

## Data Archiving Statement

Data available from the Dryad Digital Repository: http://dx.doi.org/10.5061/dryad.8b717.
